# Flavonoid glycosides and their putative human metabolites as potential inhibitors of the SARS-CoV-2 main protease (Mpro) and RNA-dependent RNA polymerase (RdRp)

**DOI:** 10.1590/0074-02760200207

**Published:** 2020-09-30

**Authors:** Felipe Moura A da Silva, Katia Pacheco A da Silva, Luiz Paulo M de Oliveira, Emmanoel V Costa, Hector HF Koolen, Maria Lúcia B Pinheiro, Antonia Queiroz L de Souza, Afonso Duarte L de Souza

**Affiliations:** 1 Universidade Federal do Amazonas, Centro de Apoio Multidisciplinar, Central Analítica, Manaus, AM, Brasil; 2 Secretaria Municipal de Saúde, Prefeitura de Itabirito, Itabirito, MG, Brasil; 3 Universidade Federal do Amazonas, Departamento de Química, Manaus, AM, Brasil; 4 Universidade do Estado do Amazonas, Grupo de Pesquisa em Metabolômica e Espectrometria de Massas, Manaus, AM, Brasil; 5 Universidade Federal do Amazonas, Faculdade de Ciências Agrárias, Manaus, AM, Brasil

**Keywords:** COVID-19, rutin, nicotiflorin, glucuronides, Dysphania ambrosioides, phytomedicines

## Abstract

**BACKGROUND:**

Since the World Health Organization (WHO) declared Coronavirus disease 2019 (COVID-19) to be a pandemic infection, important severe acute respiratory syndrome coronavirus 2 (SARS-CoV-2) non-structural proteins (nsp) have been analysed as promising targets in virtual screening approaches. Among these proteins, 3-chymotrypsin-like cysteine protease (3CLpro), also named main protease, and the RNA-dependent RNA polymerase (RdRp), have been identified as fundamental targets due to its importance in the viral replication stages.

**OBJECTIVES:**

To investigate, *in silico*, two of the most abundant flavonoid glycosides from *Dysphania ambrosioides*; a medicinal plant found in many regions of the world, along with some of the putative derivatives of these flavonoid glycosides in the human organism as potential inhibitors of the SARS-CoV-2 3CLpro and RdRp.

**METHODS:**

Using a molecular docking approach, the interactions and the binding affinity with SARS-CoV-2 3CLpro and RdRp were predicted for quercetin-3-*O*-rutinoside (rutin), kaempferol-3-*O*-rutinoside (nicotiflorin) and some of their glucuronide and sulfate derivatives.

**FINDINGS:**

Docking analysis, based on the crystal structure of 3CLpro and RdRp, indicated rutin, nicotiflorin, and their glucuronide and sulfate derivatives as potential inhibitors for both proteins. Also, the importance of the hydrogen bond and π-based interactions was evidenced for the presumed active sites.

**MAIN CONCLUSIONS:**

Overall, these results suggest that both flavonoid glycosides and their putative human metabolites can play a key role as inhibitors of the SARS-CoV-2 3CLpro and RdRp. Obviously, further researches, mainly *in vitro* and *in vivo* experiments, are necessary to certify the docking results reported here, as well as the adequate application of these substances. Furthermore, it is necessary to investigate the risks of *D. ambrosioides* as a phytomedicine for use against COVID-19.

Since the appearance of the first cases, reported in December 2019 in Wuhan, China, the new pandemic disease Coronavirus disease 2019 (COVID-19) caused by severe acute respiratory syndrome coronavirus 2 (SARS-CoV-2) has claimed millions of victims and caused hundreds of deaths around the world. Forthwith, Chinese teams sequenced SARS-CoV-2^1^ and its important nonstructural proteins (nsp) were revealed, which included spike protein, 3-chymotrypsin-like cysteine protease (3CLpro), also named main protease (Mpro), papain-like protease (PLpro), and RNA-dependent RNA polymerase (RdRp).^2^ The spike protein binds the virus to the human receptor - a metallopeptidase named angiotensin-converting enzyme 2 (ACE2), while the 3CLpro and the PLpro provide components for packaging new virions from large viral polyproteins translated on host ribosome, and finally the RdRp replicate the SARS-CoV-2 RNA genome.^2^ Due to their importance in the viral replication stages, 3CLpro and RdRp have been highlighted as fundamental targets in computational strategies, such as molecular docking.^3^ ^,^ ^4^ ^,^ ^5^ ^,^ ^6^ Nowadays, molecular docking represents a powerful, rational and low-cost tool, which allows understanding how these important nsps interacts with ligands at the active site, thus supporting the design and screening of new antiviral agents against COVID-19.^3^ ^,^ ^4^ ^,^ ^5^ ^,^ ^6^ Regarding the ligands, drugs used against other human diseases and natural products present in medicinal plants, such as flavonoids, have been the main candidates in virtual screening approaches.^3^ ^,^ ^4^ ^,^ ^5^ ^,^ ^6^ On the other hand, since many people have almost none access to medicines, including indigenous tribes and riverside dwellers in Amazon, medicinal plants could be the only possibility of treatment. Thus, the study of these therapeutic plants is a matter of human solidarity and can play an important role in saving lives. *Dysphania ambrosioides* (L.) Mosyakin & Clemants (Syn. *Chenopodium ambrosioides* L.) is one medicinal plants commonly found in tropical and subtropical regions and, therefore, accessible as therapeutic agents. *D. ambrosioides* is popularly known in Brazil as “mastruz” or “Erva-de-Santa-Maria” and has been used to treat a number of health problems, such as infections, sinusitis, gastritis, inflammations, and flu.^7^ ^,^ ^8^ From the phytochemical viewpoint, this species is a promising source of flavonoid glycosides, such as rutin, nicotiflorin, and other quercetin and kaempferol derivatives.^9^ These flavonoids present great biological potential, including antioxidant and antiviral activities, and some are mentioned as potential substances against Covid-19.^4^ ^,^ ^5^ ^,^ ^10^ Since *D. ambrosioides* and several other flavonoid-producing sources are used worldwide, the absorption, metabolism, and pharmacokinetics of flavonoids have been intensively investigated, and glucuronide and sulfates can be highlighted as important metabolite products in this process.^11^ ^,^ ^12^ Thus, in the present study, we screened rutin and nicotiflorin, two of the most abundant flavonoid glycosides from *D. ambrosioides*, along with some of their putative derivatives in the human organism using molecular docking, in order to test them as potential inhibitors of the SARS-CoV-2 3CLpro and RdRp.

## MATERIALS AND METHODS

**Fig. 1:**
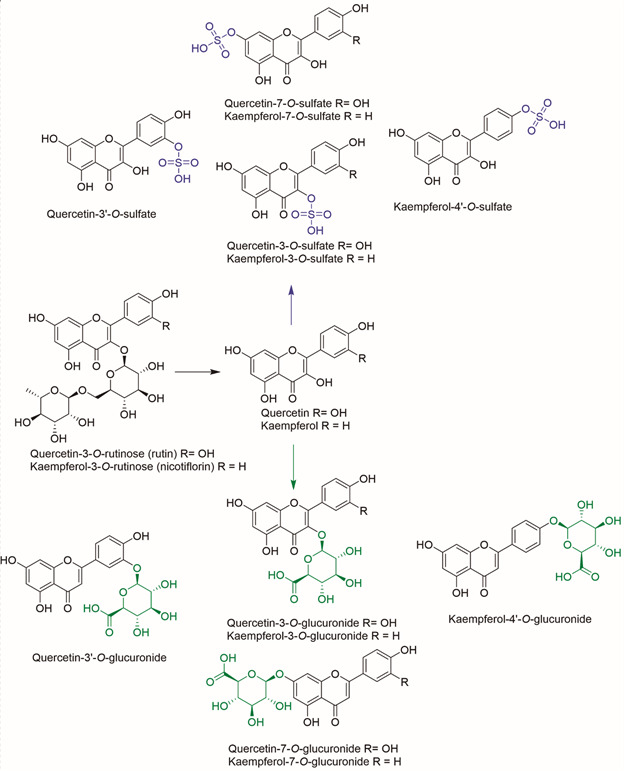
chemical structures of rutin and nicotiflorin, along with some of their putative derivatives in human organism, including sulfate (blue) and glucuronide (green) derivatives.

*Ligand preparation* - Initially, the three-dimensional (3D) structures of quercetin-3-*O*-rutinoside (rutin), quercetin-3-*O*-glucuronide, quercetin-3’-*O*-sulfate, and quercetin were downloaded from ZINC database (http://zinc15.docking.org/) in spatial data file (SDF) format. These structures were used as templates to generate, via Marvin Sketch software (https://chemaxon.com/products/marvin), kaempferol-3-*O*-rutinoside (nicotiflorin), kaempferol, and some of their putative mono-glucuronide and sulfate derivatives (Fig. 1), based on previous pharmacokinetic studies on the human organism.^11^ ^,^ ^12^ Also, theaflavin, a phenolic compound suggested as potential SARS-CoV-2 RdRp inhibitor,^13^ was downloaded from ZINC database. All explicit hydrogens were added into the structures using Marvin Sketch and the compounds were saved in SDF format. Subsequently, all the structures were subjected to geometry optimisation by the semi-empirical method PM7 using MOPAC2016 software (http://openmopac.net/MOPAC2016.html), being the results saved in protein data bank (PDB) format. Finally, the ligands were prepared for molecular docking using AutoDock Tools.^14^ Briefly, Gasteiger charges were added for each compound and non-polar hydrogens were merged, being the results saved in protein data bank, partial charge (Q), & Atom Type (T) (PDBQT) format.

**Fig. 2:**
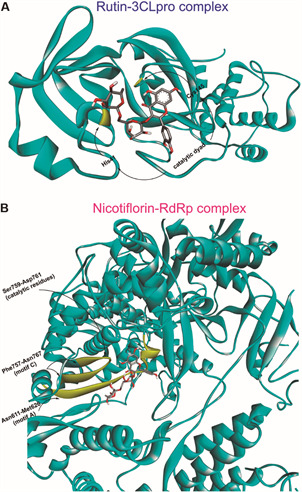
3D representation of rutin-3CLpro complex (A) and nicotiflorin-RdRp complex (B), highlighting key residues in the receptor-binding pockets.

*Protein preparation* - The 3D crystal structures of the SARS-CoV-2 3CLpro (PDB ID: 6W63) and RdRp (PDB ID: 6M71) were retrieved from Research Collaboratory for Structural Bioinformatics Protein Data Bank (RCSB PDB) (http://www.rcsb.org) in PDB format. These receptors were prepared using AutoDock Tools. Briefly, water molecules and bound ligands were removed, polar hydrogens and Kollman charges were added, and the non-polar hydrogens were merged. For both proteins, the protonation states of the amino acid residues were automatically generated by Autodock tools [Supplementary data (Figs 1-2)] based on the protonation states of the original 3D crystal structures. Finally, the results were saved as PDBQT format.

*Docking simulations* - The docking simulations were as previously reported,^15^ in which the grid box was centered at the ligand X77 in 3CLpro (PDB ID: 6W63) and at the presumed active site^16^ in the RdRp (PDB ID: 6M71). For 3CLpro, the grid box was centered at x = -20.810, y = 19.141, and z = -29.186, with x = 27 Å, y = 25 Å, and z = 25 Å size. On the other hand, the RdRp grid box was centered at the x = 117.382, y = 111.853, and z = 121.073, with x = 22 Å, y = 30 Å, and and z = 46 Å size. The interactions and the binding affinity of the protein-ligand complex were predicted via a docking process using Autodock Vina, which use a Broyden-Fletcher-Goldfarb-Shanno (BFGS) algorithm, through an Iterated Local Search method, to generate different ligand conformers.^17^ Regarding scoring function, Autodock Vina uses a hybrid score function that combine empirical and knowledge-based functions.^17^ Finally, the results were viewed with the Discovery Studio software.^18^ Due to the lack of models with ligands for RdRp in the RCSB PDB, only the 3CLpro was tested for redocking.

## RESULTS

*Molecular docking with 3CLpro (main protease* - *Mpro)* - Docking analysis applied to the 3CLpro protein revealed close scoring function values for rutin (-9.2 kcal/mol), nicotiflorin (-8.9 kcal/mol), and the previously described inhibitor X77 (redocking binding free energy = -8.4 kcal/mol, RMSD = 0.8909 Å), which suggests the establishment of favorable interactions for the ligand-3CLpro complex. Moreover, glucuronide derivatives from rutin (-8.4 to -8.5 kcal/mol) and nicotiflorin (-8.0 to -8.3 kcal/mol) presented binding free energies similar to X77. Also, the binding free energies for all sulfate derivatives (-8.1 to 8.4 kcal/mol), except 3-*O*-sulfates (-7.3 to -7.6 kcal/mol), were close to value for X77. By their turn, quercetin (-7.5 kcal/mol) and kaempferol (-7.2 kcal/mol) presented similar binding free energies to 3-*O*-sulfates derivatives.

**Fig. 3:**
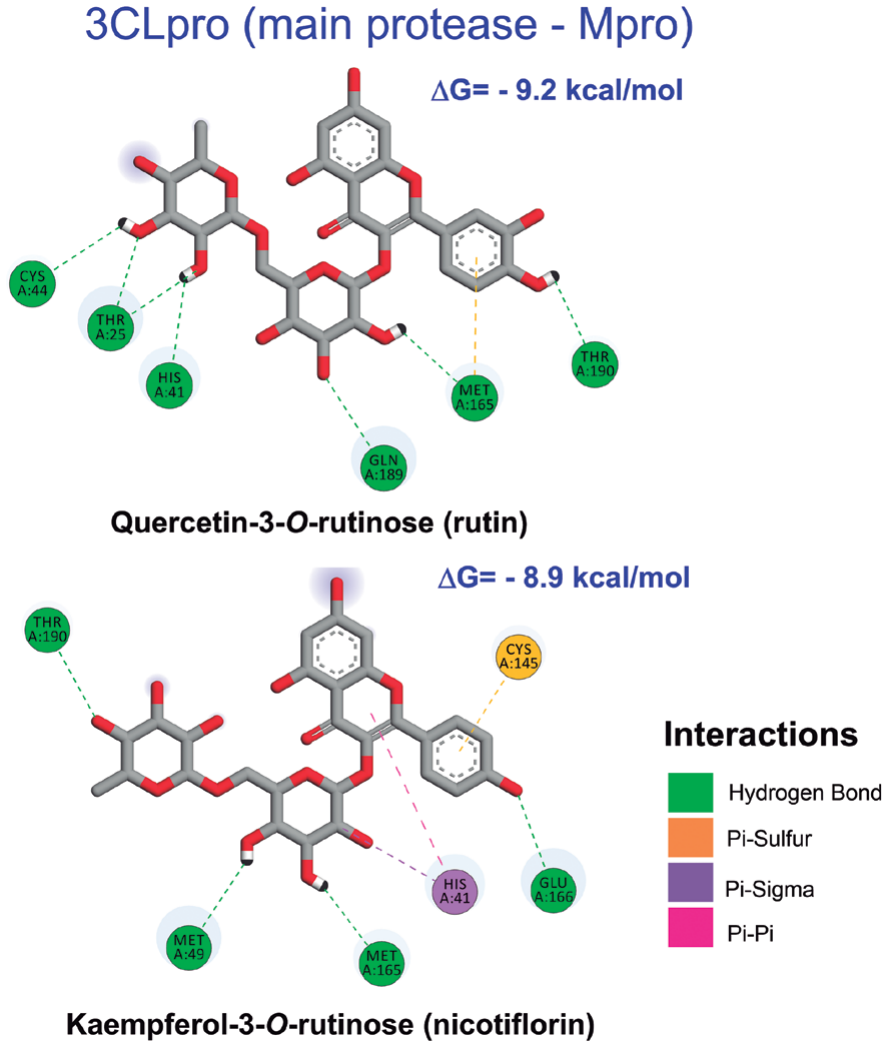
main interactions observed for the top-scored inhibitors of 3CLpro by docking analysis. Only hydrogen atoms that are actually participating in hydrogen bonds were presented.

**Fig. 4:**
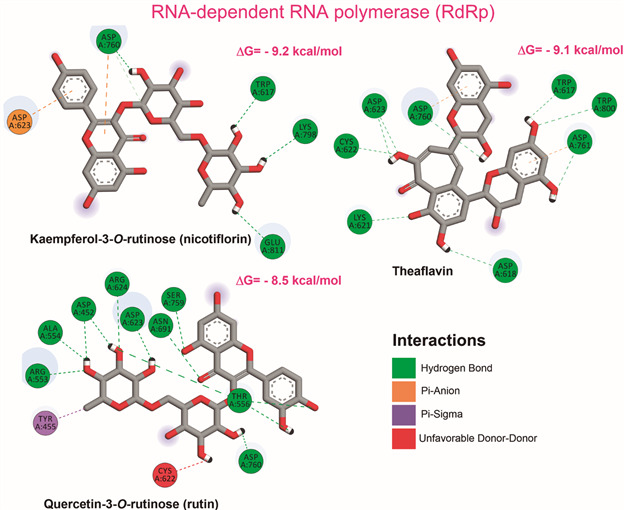
main interactions observed for theaflavin, rutin and nicotiflorin-RdRp complex by docking analysis. Only hydrogen atoms that are actually participating in hydrogen bonds were presented.

In regards to the observed interactions in the presumed active site of 3CLpro, hydrogen bonds and π-based interactions, such as π-sulfur, π-alkyl, π-π, and π-cation interactions, were dominant in almost all tested compounds (Table) [Supplementary data (Figs 3-19)]. Since recent studies demonstrated that the catalytic dyad (His41 and Cys145) in the receptor-binding pocket of SARS-CoV-2 3CLpro (Fig. 2A), such as in SARS-CoV 3CLpro, is fundamental to the proteolytic task, we considered in our interpretation the interactions of ligands with this dyad, along with that observed to x77 inhibitor.^3^ ^,^ ^5^ ^,^ ^19^ For x77, hydrogen bonds were observed with Gly143, Cys145, His163, and Glu166, along with π-sulfur interactions with Met49 and Cys145, π-alkyl interaction with Cys145, π-amide interaction with Leu141, and π-π interaction with His41 [Supplementary data (Fig. 3)].

**TABLE:** Docking analysis data for rutin and nicotiflorin, two of the most abundant flavonoid glycosides from *Dysphania ambrosioides*, along with some of their putative derivatives in human organism

Compounds	Protein (PDB iD)	Binding energy (kcal/mol)	Main interactions
Theaflavin	RdRp (6M71)	-9.1	HB (Trp617, Asp618, Lys621, Cys622, Asp623, Asp760, Asp761, Trp800), PA (Asp760, Asp761)
x77	3CLpro (6W63)	-8.4	HB (Gly143, Cys145, His163, Glu166), PS (Met49, Cys145), PAl (Cys145), PAm (Leu141), PP (His41)
Quercetin-3-*O*-rutinose (rutin)	RdRp (6M71)	-8.5	HB (Asp452, Arg553, Ala554, Thr556, Asp623, Arg624, Asn691, Ser759, Asp760), PSi (Tyr455)
	3CLpro (6W63)	-9.2	HB (Thr25, His41, Cys44, Met165, Gln189, Thr190), PS (Met165)
Quercetin-7-*O*-glucuronide	RdRp (6M71)	-8.2	HB (Arg553, Thr556, Lys621, Asp623, Asp760, Asp761), PA (Asp760)
	3CLpro (6W63)	-8.4	HB (Thr24, Thr25, Ser46, Phe140, Ser144, Glu166), PC (His41), PS (Cys145), PP (His41), PAl (Cys145)
Quercetin-3’-*O*-glucuronide	RdRp (6M71)	-8.2	HB (Asp760, Asp761, Trp617, Trp800, Glu811), PAm (Tyr619)
	3CLpro (6W63)	-8.5	HB (Thr25, His41, Cys44, Ser144, His163, Glu166), PS (Met49), PAl (Met49, Met165)
Quercetin-3-*O*-glucuronide	RdRp (6M71)	-8.0	HB (Trp617, Tyr619, Asp761, Trp800, Glu811, Cys813), PA (Asp760, Asp761), PAl (Cys813)
	3CLpro (6W63)	-8.5	HB (Glu166, Arg188, Gln189), PS (Cys145), PC (His41), PAl (Met 165)
Quercetin-7-*O*-sulfate	RdRp (6M71)	-8.0	HB (Lys545, Arg553, Ala554, Thr556, Arg624, Asn691), PC (Arg553), PAl (Arg555)
	3CLpro (6W63)	-8.4	HB (Phe140, Leu141, His163, Glu166, Thr190, Gln192), PS (Cys145), PAl (Met165)
Quercetin-3-*O*-sulfate	RdRp (6M71)	-7.1	HB (Asp760, Ser814), PA (Asp761, Glu811)
	3CLpro (6W63)	-7.6	HB( Gly143, Ser144, Glu166, Arg188), PS (Met165), PAl (Met165)
Quercetin-3’-*O*-sulfate	RdRp (6M71)	-8.1	HB (Asp452, Arg553, Thr556, Tyr619, Lys621, Arg624), PC (Arg553), PAl (Lys621)
	3CLpro (6W63)	-8.1	HB (His41, Met49, Ser144, Cys145, His163, Glu166), PC (His41), PS (Cys44, Met49, His163)
Quercetin	RdRp (6M71)	-7.4	HB (Met615, Ile779, Cys799), PSi (Ile779, Thr801)
	3CLpro (6W63)	-7.5	HB (Glu166, Asp187, Thr190, Gln192), PS (Met165), PAl (Met165, Pro168)
Kaempferol-3-*O*-rutinose (nicotiflorin)	RdRp (6M71)	-9.2	HB (Trp617, Asp760, Lys798, Glu811), PA (Asp623, Asp760)
	3CLpro (6W63)	-8.9	HB (Met49, Met165, Glu166, Thr190), PS (Cys145), PSi (His41), PP (His41)
Kaempferol-4’-*O*-glucuronide	RdRp (6M71)	-8.3	HB (Tyr619, Cys622, Asp623, Asp761, Lys798, Ser814), PC (Lys621)
	3CLpro (6W63)	-8.0	HB (Thr25, His41, Arg188, Gln192), PAl (Met49, Met165, Pro168)
Kaempferol-3-*O*-glucuronide	RdRp (6M71)	-7.9	HB (Trp617, Tyr619, Asp761, Trp800, Glu811, Cys813), PA (Asp760, Asp761), PAl (Cys622, Cys813)
	3CLpro (6W63)	-8.3	HB (Thr25, Asn142, Gly143, Ser144, His163, Glu166), PS (Met49), PP (His41), PSi (Thr25)
Kaempferol-7-*O*-glucuronide	RdRp (6M71)	-8.0	HB (Asp761, Lys798, Trp800, Ser814), PAl (Pro620, Lys798)
	3CLpro (6W63)	-8.3	HB (His41, Arg188, Gln189, Thr190), PS (Cys44, Met49), PSi (Thr25), PAl (Met49, Cys145)
Kaempferol-7-*O*-sulfate	RdRp (6M71)	-7.9	HB (Asp452, Lys545, Arg553, Ala554, Arg555, Thr556, Arg624, Asn691), PC (Arg553)
	3CLpro (6W63)	-8.3	HB (Cys44, Ser144, His163), PS (Cys145), PP (His41), PAl (Met49, Met165)
Kaempferol-4’-*O*-sulfate	RdRp (6M71)	-7.3	HB (Lys621, Thr687, Asn691, Asp760), PC (Arg553), PA (Asp623, Asp760), PAl (Lys621, Cys622)
	3CLpro (6W63)	-8.2	HB (Ser144, Glu166, Asp187, Gln189), PAl (Met165)
Kaempferol-3-*O*-sulfate	RdRp (6M71)	-6.7	HB (Ser814), PA (Asp761, Glu811)
	3CLpro (6W63)	-7.3	HB (Glu166, Asp187, Gln189), PS (Met49), PAl (Cys145)
Kaempferol	RdRp (6M71)	-7.2	HB (Ser778, Glu796), PSi (Ile779, Thr801)
	3CLpro (6W63)	-7.2	HB (Glu166), PS (Cys44, Met165), PAl (Met49, Pro168)

HB: hydrogen bond; PS: π-sulfur; Pal: π-alkyl; PP: π-π; PA: π-anion; PC: π-cation; Psi: π-sigma; Pam: π-amide.

For rutin, the top-scored compound for 3CLpro, several hydrogen bonds were observed, including a key interaction with His41, from the catalytic dyad, and interactions with Thr25, Cys44, Met165, Gln189, and Thr190 (Fig. 3). The observed hydrogen bonds with His41 and Thr190 are in agreement with molecular docking results previously reported to other 3CLpro crystallographic models (PDB ID: 6Y84 and 6LU7).^5^ ^,^ ^20^ On the other hand, nicotiflorin presented key interactions with both residues of the catalytic dyad of 3CLpro (His41 and Cys145) (Fig. 3), along with key residues observed for x77. In addition to hydrogen bonds with key residues such as Met49, Glu166, and Thr190, this compound presented π-π and π-sigma interactions with His41, and π-sulfur interaction with Cys145 from the catalytic dyad. Key interactions with His41 or Cys145 were also dominant in almost all glucuronide and sulfate derivatives. For example, quercetin-3’-*O*-glucuronide, quercetin-3’-*O*-sulfate, kaempferol-7-*O*-glucuronide, and kaempferol-4’-*O*-glucuronide presented hydrogen bonds with His41, while quercetin-7-*O*-glucuronide, quercetin-3-*O*-glucuronide, quercetin-7-*O*-sulfate, and kaempferol-7-*O*-sulfate presented π-sulfur interaction with Cys145. The main observed interactions between these compounds and 3CLpro are summarised in Table.

*Molecular docking for RNA-dependent RNA polymerase (RdRp)* - Docking analysis applied to the RdRp protein revealed scoring function values close for nicotiflorin (-9.2 kcal/mol), rutin (-8.5 kcal/mol), and theaflavin (-9.1 kcal/mol) (Table). Similarly to the 3CLpro molecular docking, glucuronide derivatives (quercetin = -8.0 to -8.2 kcal/mol; kaempferol = -7.9 to -8.3 kcal/mol) presented close binding free energies to the sulfate derivatives (quercetin = -8.0 to -8.1 kcal/mol; kaempferol-7-*O*-sulfate = -7.9 kcal/mol), except kaempferol-3-*O*-sulfate (-6.7 kcal/mol), quercetin-3-*O*-sulfate (-7.1 kcal/mol), and kaempferol-4’-*O*-sulfate (-7.3 kcal/mol). Also, quercetin (-7.4 kcal/mol) and kaempferol (-7.2 kcal/mol) presented similar minor binding free energies.

Regarding to the observed interactions in the presumed active site of RdRp, hydrogen bonds, π-cation and π-anion interactions were dominant to almost all tested compounds (Table) [Supplementary data (Figs 20-36)]. Since this active site includes the motif A (residues 611-626), with the classic divalent cation-binding residue 618, and motif C (residues 753-767), with the catalytic residues 759-761,^16^ (Fig. 2B) we therefore focused our interpretation on these interactions.

The observed interactions for theaflavin were near to that previously described in the literature,^13^ and highlighted several hydrogen bonds, including that with Lys621, Cys622, and Asp623, from motif A, and that with Asp760 and Asp761, from motif C (Fig. 4), which is suggestive of a good approximation of the present model with the previous model by homology.^13^ Also, π-anion interactions were observed with the catalytic residues Asp760 and Asp761 from motif C. For nicotiflorin, the top-scored compound for RdRp, hydrogen bond and π-anion interactions were observed with the catalytic residue Asp760, from motif C (Fig. 4). In addition, hydrogen bonds with Trp617, along with π-anion interaction with Asp623, from motif A, were also observed to this compound. Also, key interactions were observed to rutin, highlighting hydrogen bonds with Asp623 and Arg624, from motif A, and with the catalytic residue Ser759 and Asp760, from motif C (Fig. 4). Moreover, several glucuronide and sulfate derivatives, such as quercetin-7-*O*-glucuronide, quercetin-3-*O*-glucuronide, quercetin-3-*O*-sulfate, kaempferol-3-*O*-glucuronide, and kaempferol-4’-*O*-sulphate, presented key hydrogen bonds and π-anion interaction with the catalytic residue from motif C. The main observed interactions between these compounds and RdRp are summarised in Table.

## DISCUSSION

Rutin and nicotiflorin, as well as other glycoside flavonoids, are present in a large number of therapeutic medicinal plants and are often consumed in the form of herbal teas.^21^ These compounds are vital in diets and are of great interest due their antioxidant, anti-inflammatory and antiviral activities and their human metabolites, which include quercetin from rutin hydrolysis.^10^ ^,^ ^21^ ^,^ ^22^ ^,^ ^23^ ^,^ ^24^ For this reason, their absorption, metabolism, toxicity, and pharmacokinetics has been intensively investigated.^11^ ^,^ ^12^ Regarding these studies, rutinoside flavonoids, such as rutin and nicotiflorin, are deglycosylated prior to being absorbed into the circulation and then conjugated mainly with glucuronate and sulfate, these being the main forms in plasma.^11^ Based on this information, it is reasonable to suggest that both quercetin and kaempferol sulfates and glucuronides, demonstrated above as promising SARS-CoV-2 3CLpro and RdRp inhibitors, could have a key role against these proteins, since the virus is also dominant in plasma. This hypothesis is in accordance with previous *in vivo* antiviral studies performed with rutin. These studies indicate that rutin protects cells for about 24 h against vesicular stomatitis virus, affords immense viral embarrassment in canine distemper virus, and demonstrates a profound antiviral effect against avian influenza strain H5N1.^10^ Flavonoid glucuronides have also been described as antiviral agents, including quercetin-3-*O*-glucuronide, here described as a potential 3CLpro inhibitor.^25^ ^,^ ^26^ Moreover, previous works have pointed out the effective anti-HIV and anti-HSV activities of flavonoid sulfates.^27^

Recent autopsy studies have permitted Brazilian COVID-19 patients to be treated as sufferers of Dissemination Intravascular of Coagulum (DIC), which can cause failure of several organs, mainly the lungs.^28^ As a preliminary stage for combating the disease, Low Molecular Weight Heparin (LMWH) has been successfully administered.^29^ In addition to its anticoagulant therapeutic effects, LMWH has demonstrated anti-inflammatory effects, endothelial protection and viral inhibition.^30^ Rutin, displayed here as an active agent against 3CLpro and RdRp of SARS-CoV-2, has also proved to have anticoagulant therapeutic effects^31^ as well as anti-inflammatory effects and potential protection against acute lung injury (ALI).^32^ Intravenous or intranasal administration could be an alternative to oral intake, thus improving its bioavailability.^33^ ^,^ ^34^ *D. ambrosioides* has been used successfully by the riverside population in the Amazon Region for treating cases of acute respiratory distress syndrome (ARDS) and tuberculosis.^8^ These results may also be related to the presence of rutin.

Overall, these results suggest that rutin, nicotiflorin, and their putative human metabolites, can play a key role as inhibitors of the SARS-CoV-2 3CLpro and RdRp. Such derivatives, which are expected in plasma, are in fact the most likely compounds for targeting these viral proteins, via oral intake of these flavonoid glycosides. However, at least for rutin, intravenous or intranasal administration can be an alternative for prompt bioavailability, without risk of digestive degradation. Our results, and much of the reported data, suggest rutin and nicotiflorin as possible alternatives for combating the COVID-19 virus. Rutin could even be considered an alternative to LMWH, given its anticoagulant and anti-inflammatory effects and its potential protection against ALI. Obviously, further researches, mainly *in vitro* and *in vivo* experiments, are necessary to corroborate the docking results reported here, as well as the adequate application of these substances. Furthermore, it is necessary to investigate the risks of *D. ambrosioides* as a phytomedicine for use against COVID-19.
