# Supplementary material for: Flavonoid glycosides and their putative human metabolites as potential inhibitors of the SARS-CoV-2 main protease (Mpro) and RNA-dependent RNA polymerase (RdRp)
Source: Mem Inst Oswaldo Cruz. 2020 Sep 30;115:e200207. doi: 10.1590/0074-02760200207 (PMC7534957; doi:10.1590/0074-02760200207)
Supplement: Supplementary file 1 [file 1678-8060-mioc-115-e200207-s.pdf]

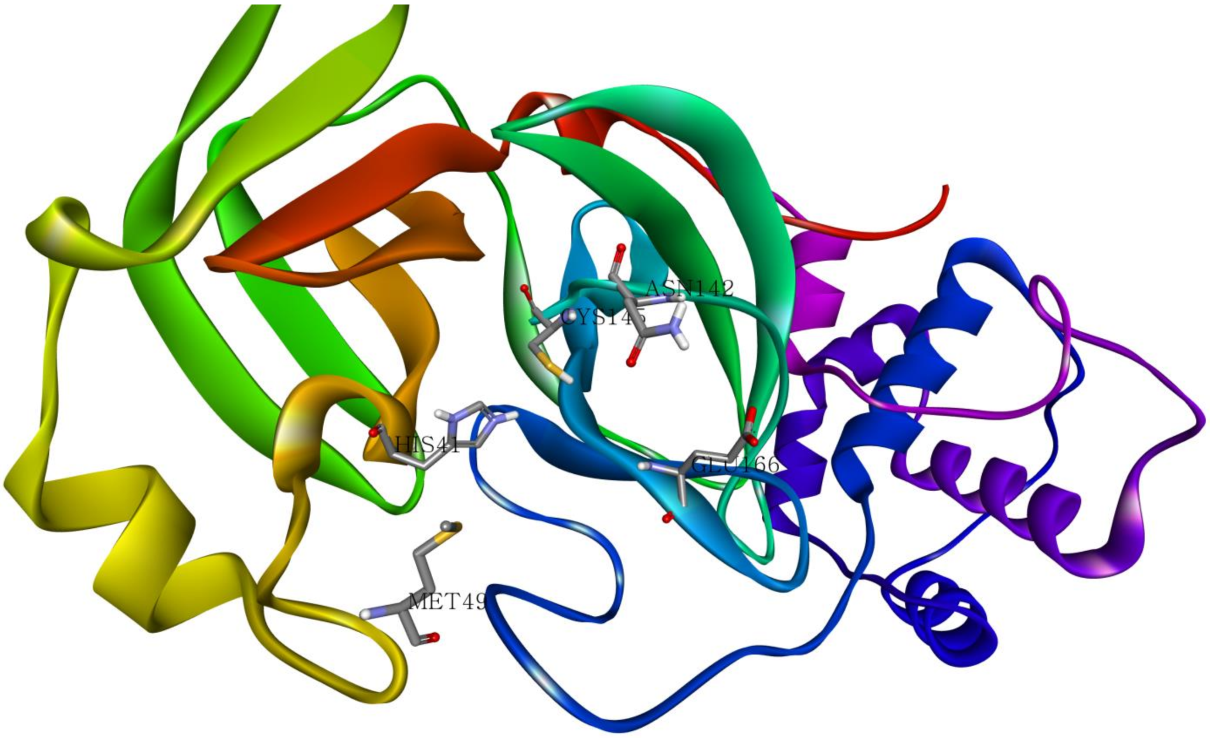

Fig. 1: protonation states of the key residues at the 3CLpro binding site.

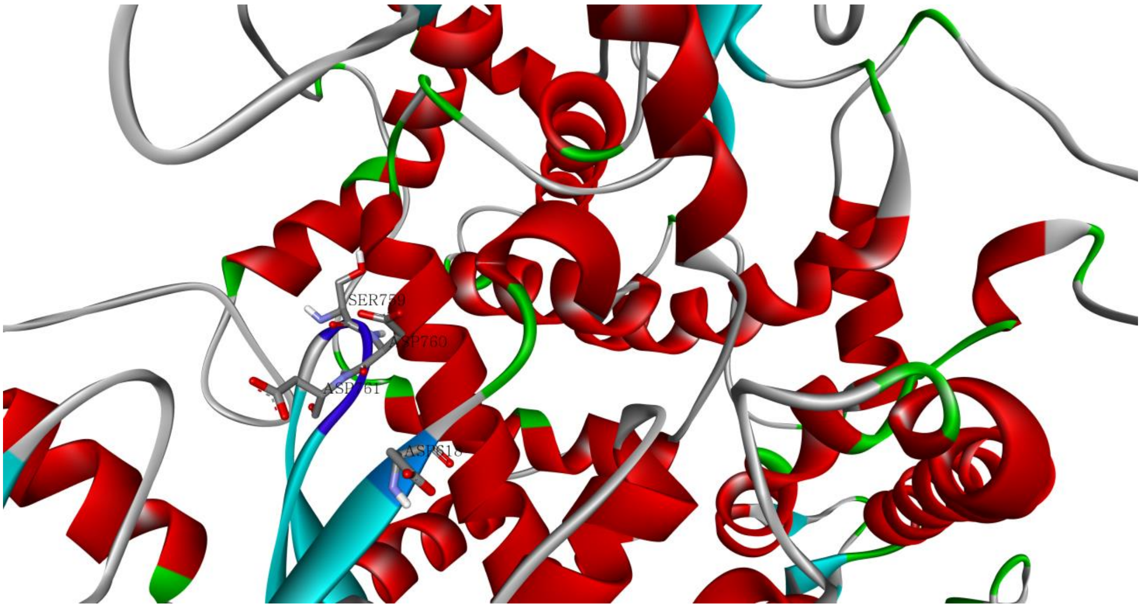

Fig. 2: protonation states of the key residues at the RdRp binding site.

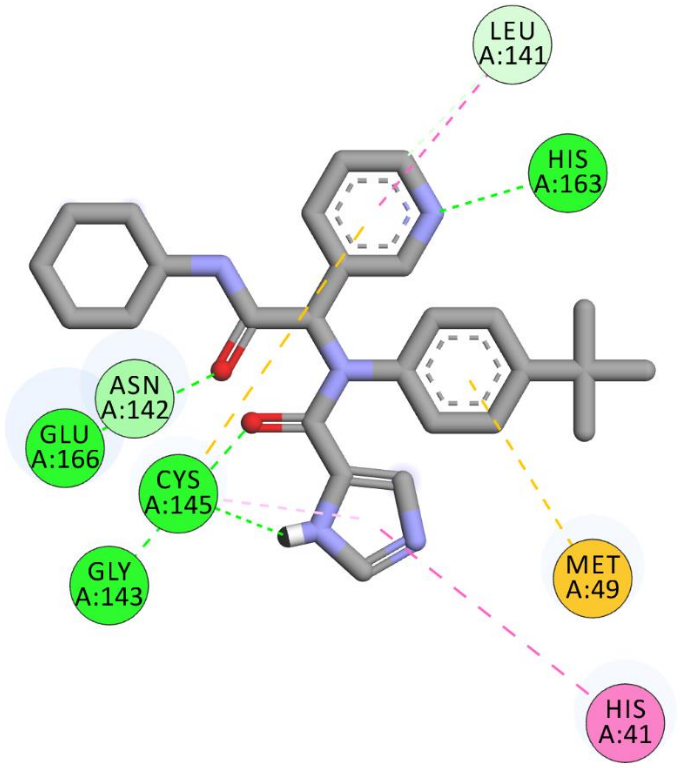

Fig. 3: main interactions observed for X77-3CLpro complex by docking analysis.

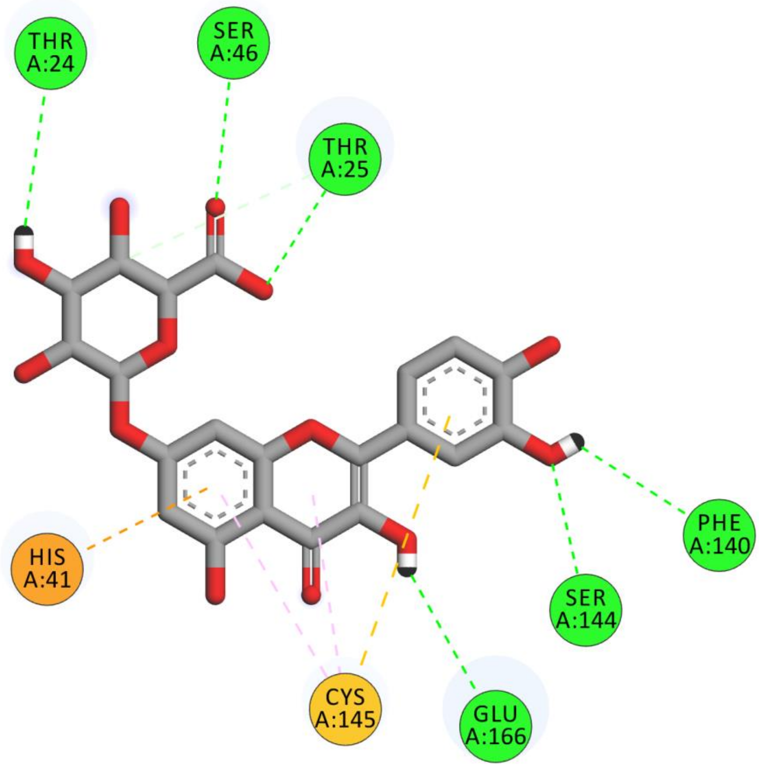

Fig. 4: main interactions observed for quercetin-7-*O*-glucuronide-3CLpro complex by docking analysis.

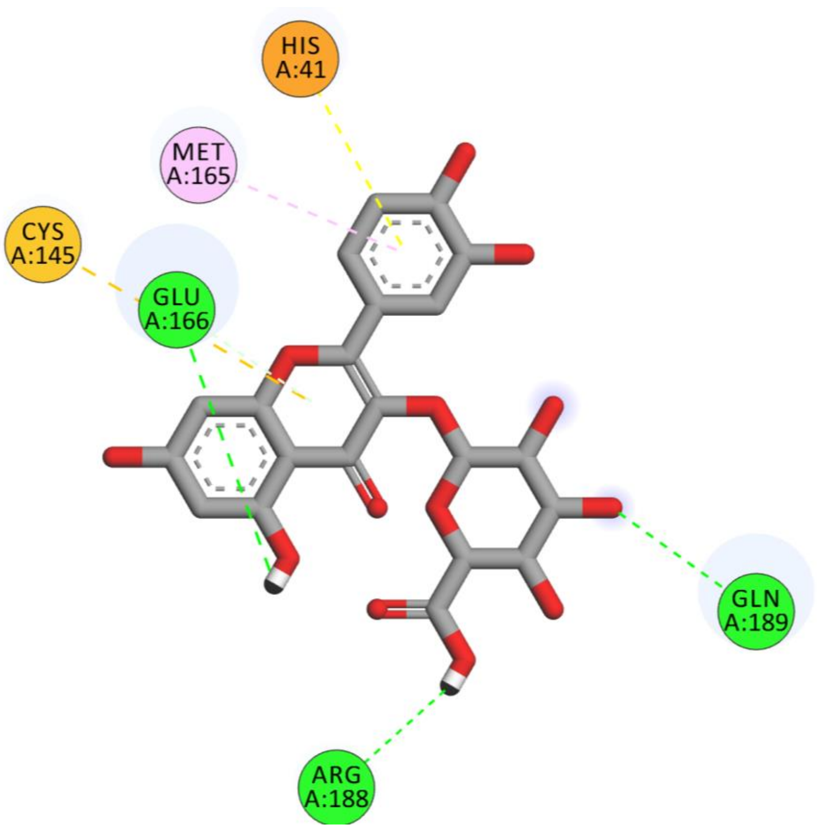

Fig. 5: main interactions observed for quercetin-3-*O*-glucuronide-3CLpro complex by docking analysis.

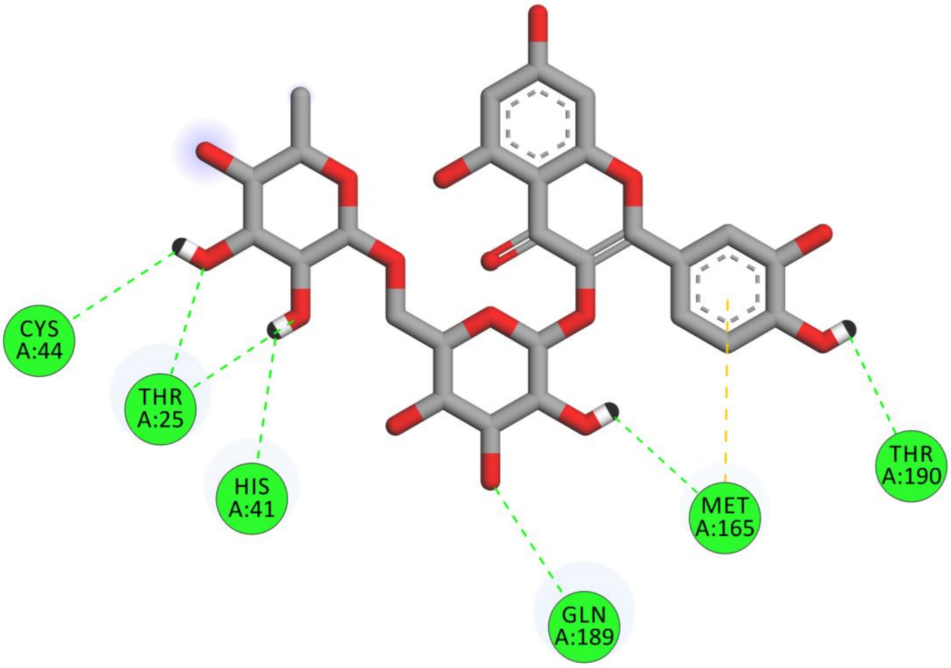

Fig. 6: main interactions observed for quercetin-3-*O*-rutinose (Rutin)-3CLpro complex by docking analysis.

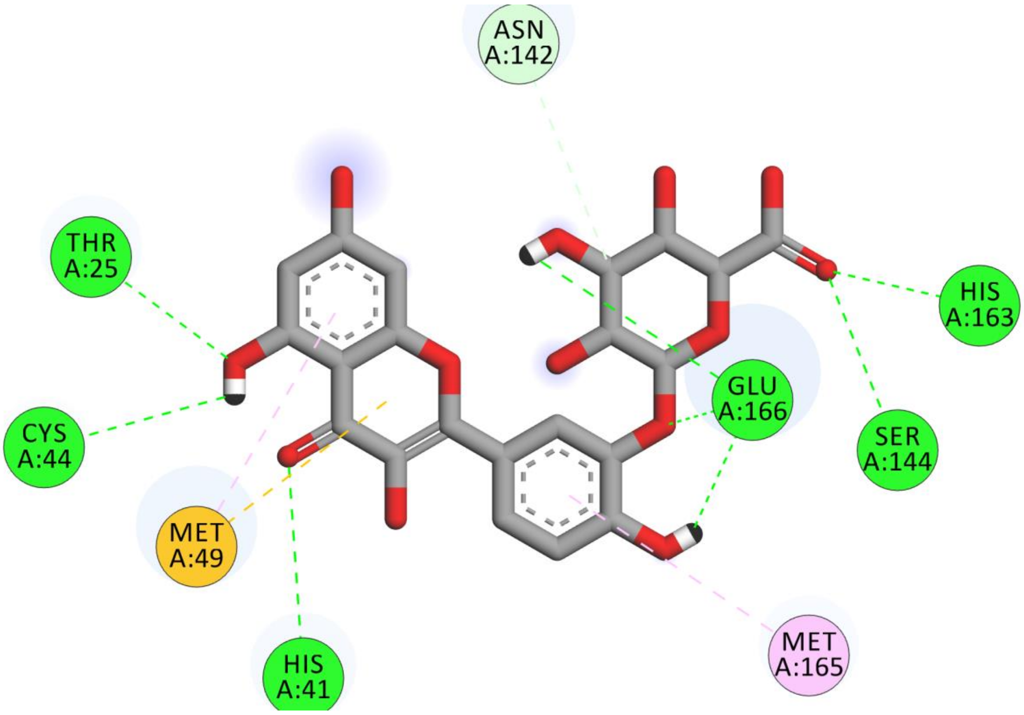

Fig. 7: main interactions observed for quercetin-3'-*O*-glucuronide-3CLpro complex by docking analysis.

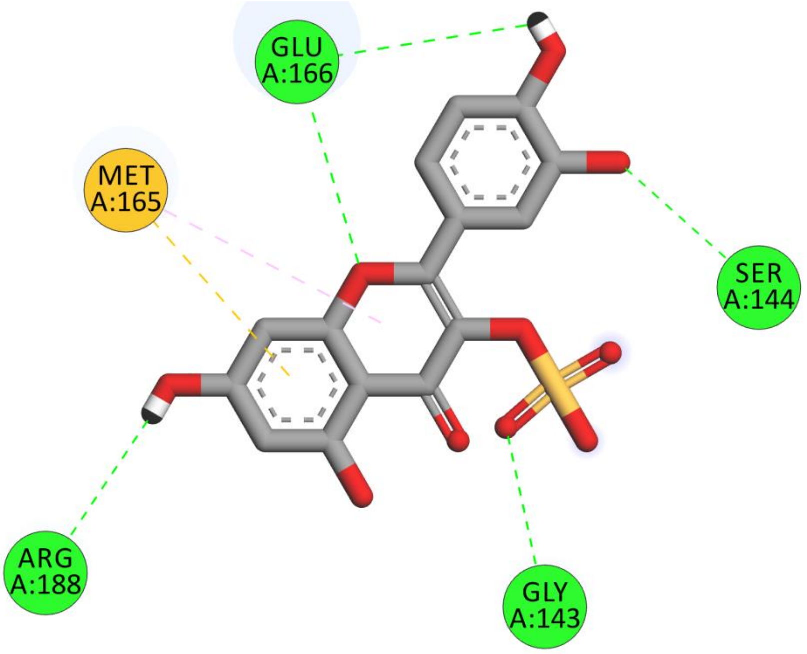

Fig. 8: main interactions observed for quercetin-3-*O*-sulphate-3CLpro complex by docking analysis.

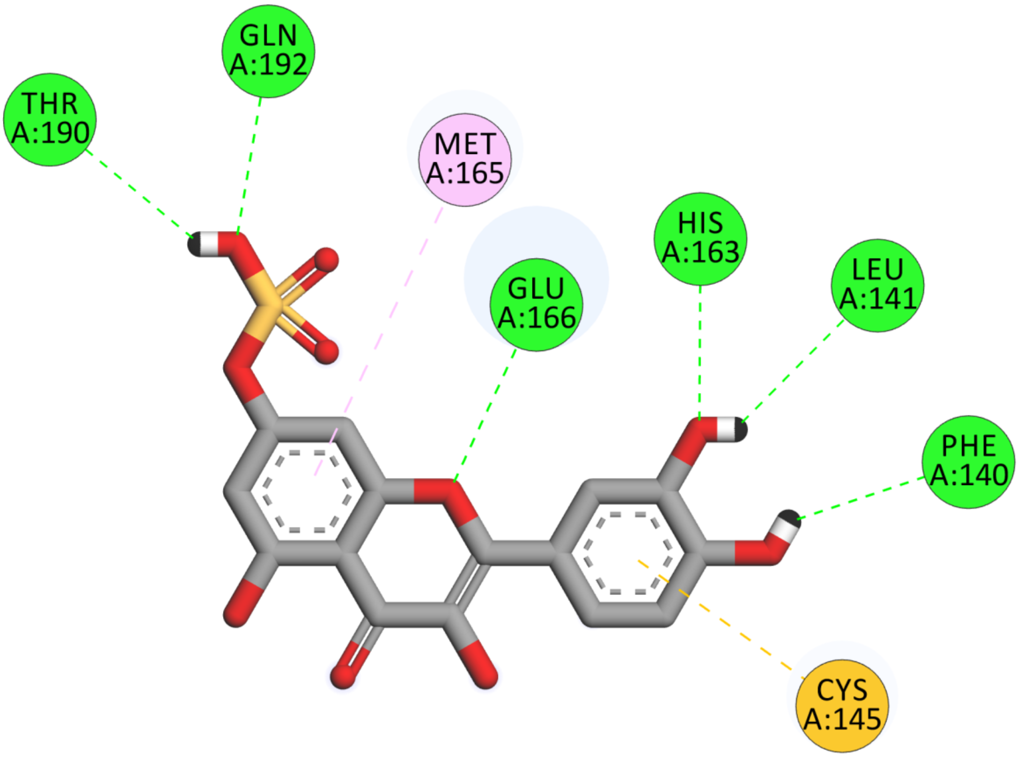

Fig. 9: main interactions observed for quercetin-7-*O*-sulphate-3CLpro complex by docking analysis.

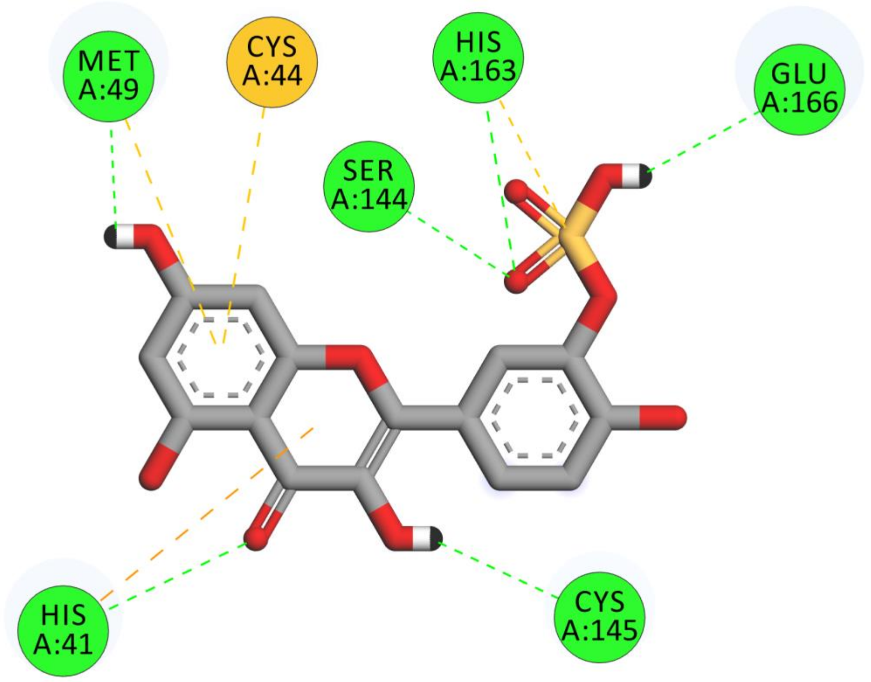

Fig. 10: main interactions observed for quercetin-3'-*O*-sulphate-3CLpro complex by docking analysis.

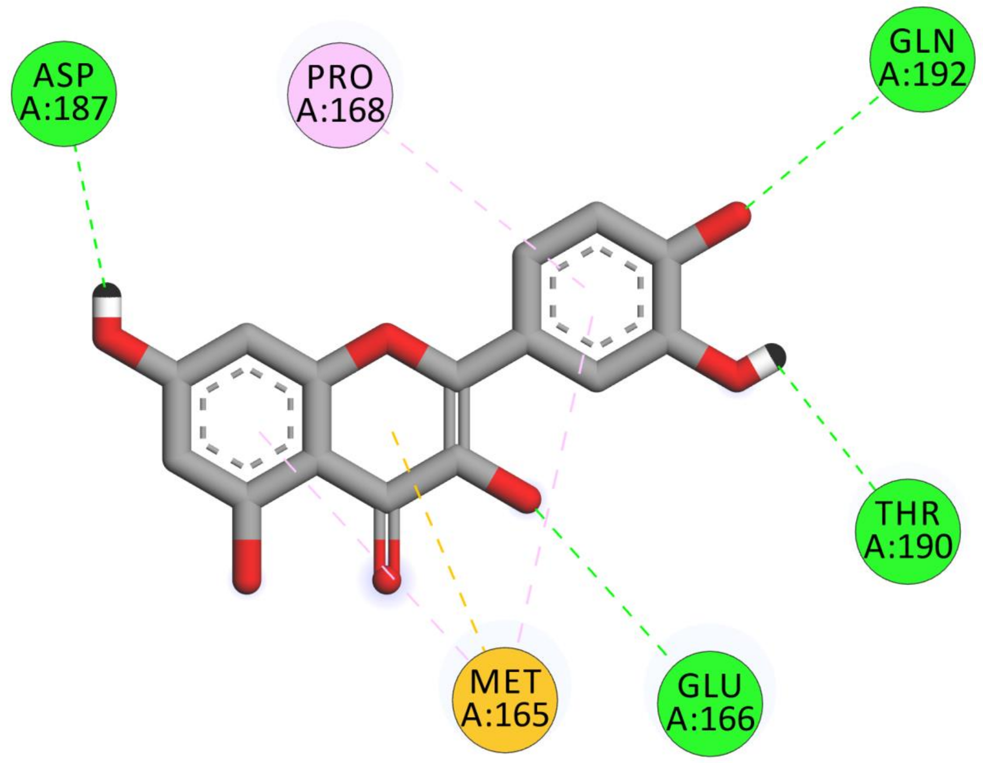

Fig. 11: main interactions observed for quercetin-3CLpro complex by docking analysis.

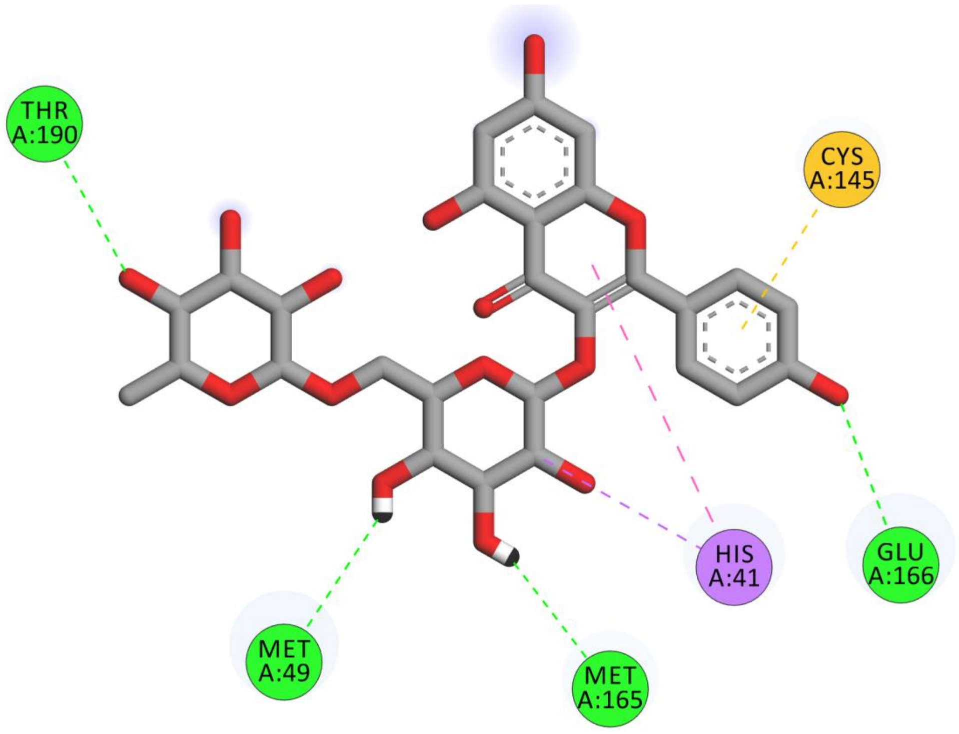

Fig. 12: main interactions observed for kaempferol-3-*O*-rutinose (Nicotiflorin)-3CLpro complex by docking analysis.

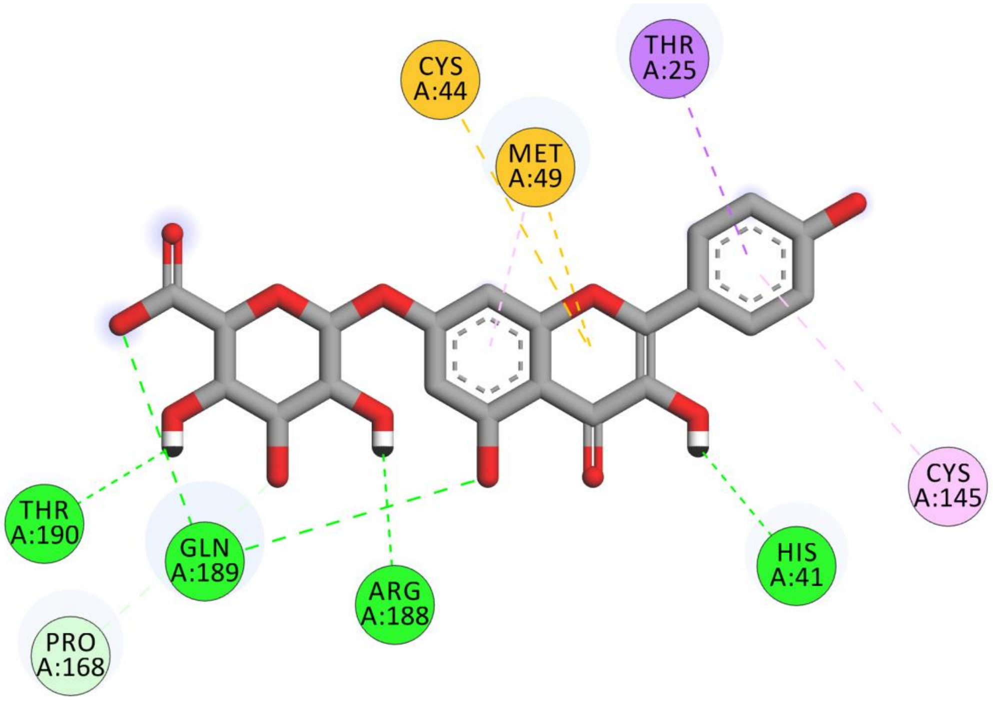

Fig. 13: main interactions observed for kaempferol-7-*O*-glucuronide-3CLpro complex by docking analysis.

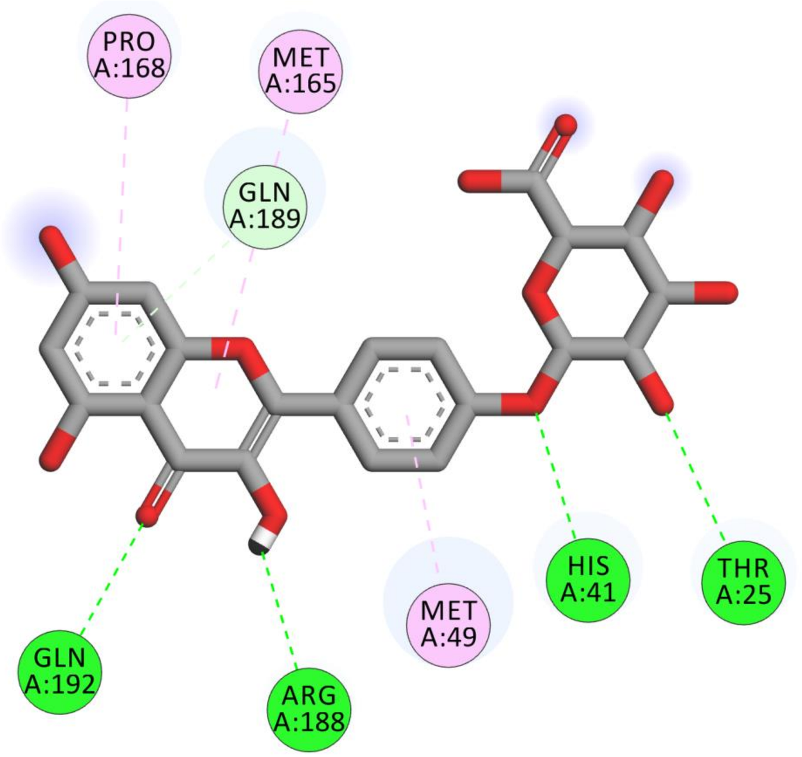

Fig. 14: main interactions observed for kaempferol-4'-*O*-glucuronide-3CLpro complex by docking analysis.

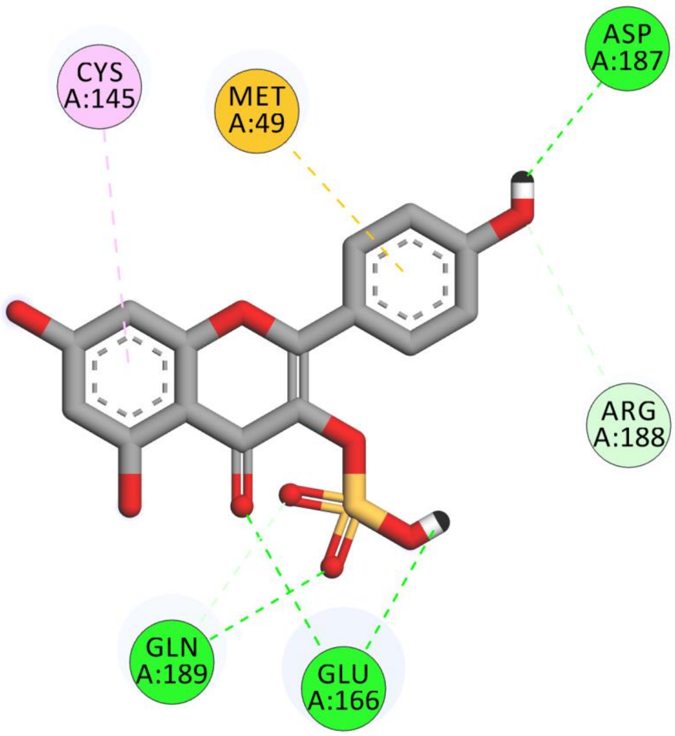

Fig. 15: main interactions observed for kaempferol-3-*O*-sulphate-3CLpro complex by docking analysis.

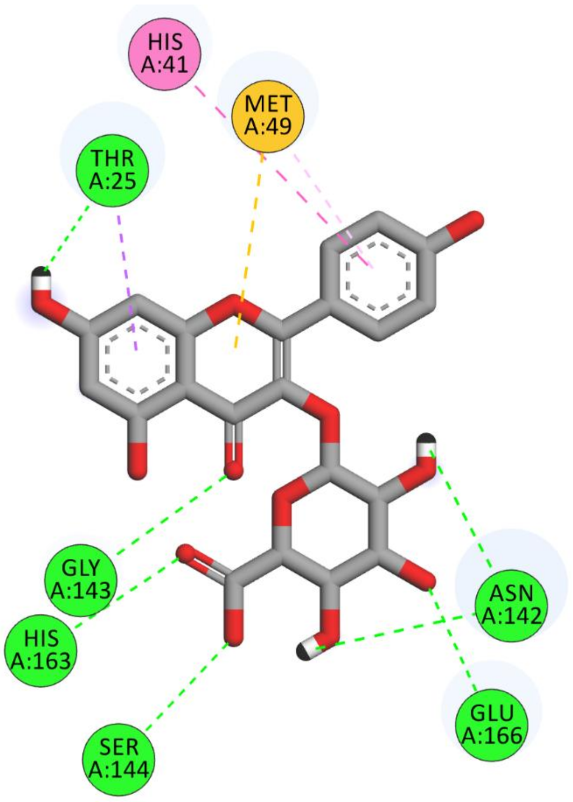

Fig. 16: main interactions observed for kaempferol-3-*O*-glucuronide-3CLpro complex by docking analysis.

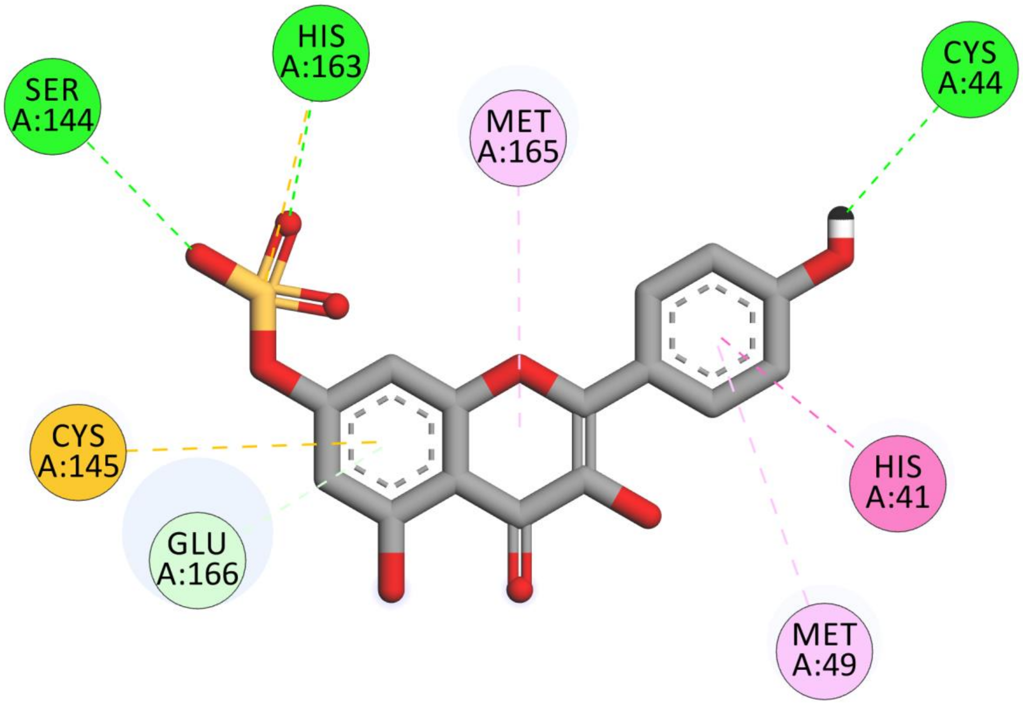

Fig. 17: main interactions observed for kaempferol-7-*O*-sulphate-3CLpro complex by docking analysis.

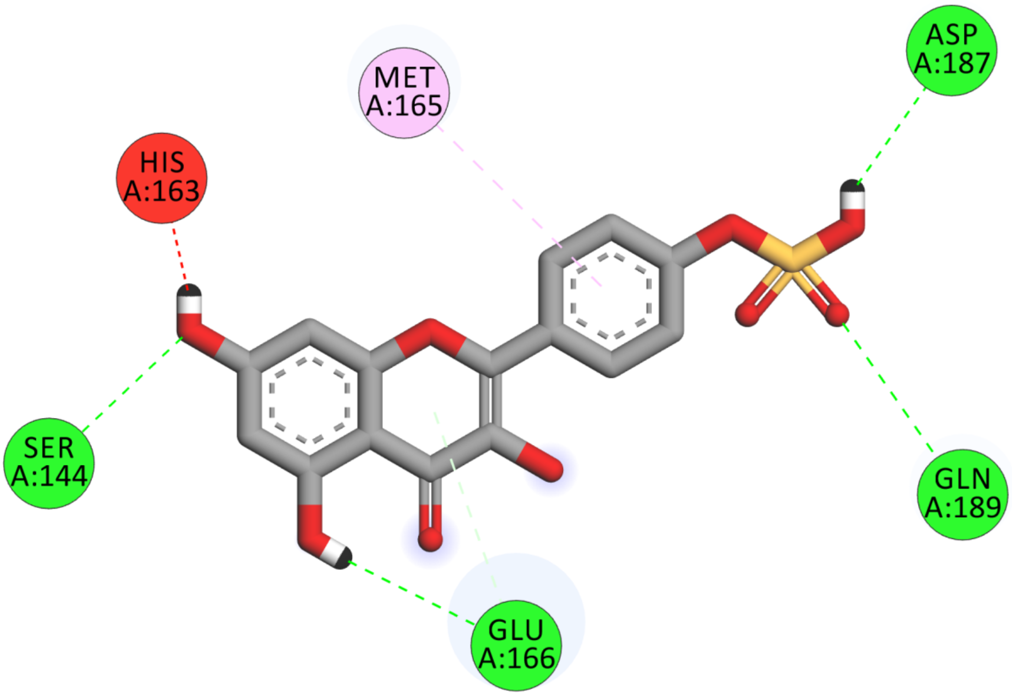

Fig. 18: main interactions observed for kaempferol-4'-*O*-sulphate-3CLpro complex by docking analysis.

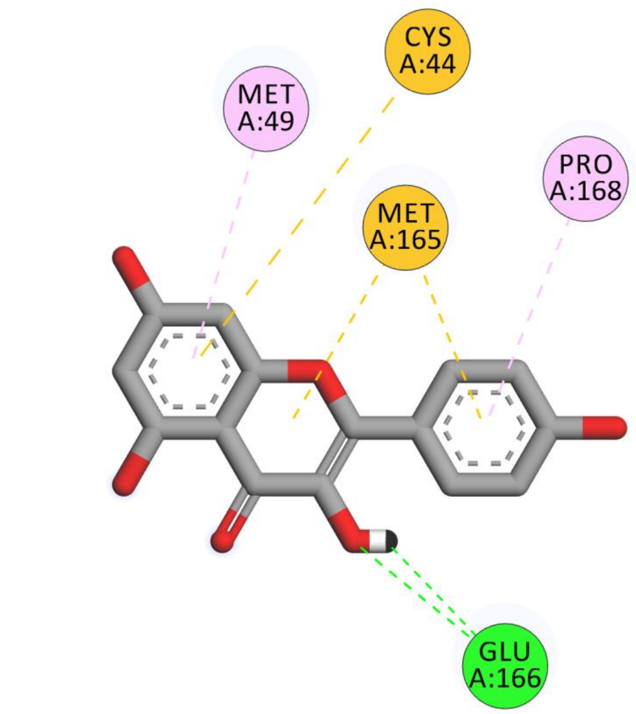

Fig. 19: main interactions observed for kaempferol-3CLpro complex by docking analysis.

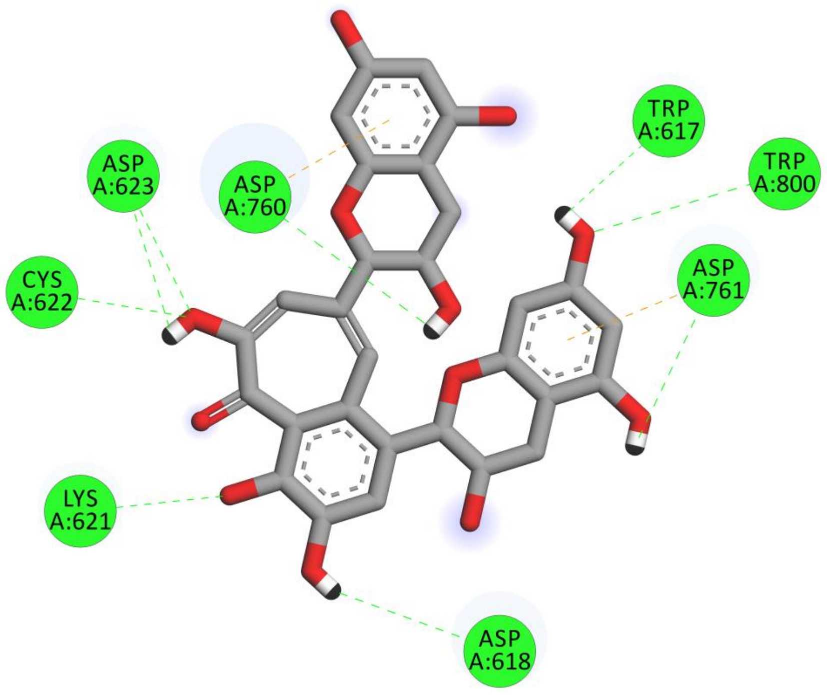

Fig. 20: main interactions observed for theaflavine-RdRp complex by docking analysis.

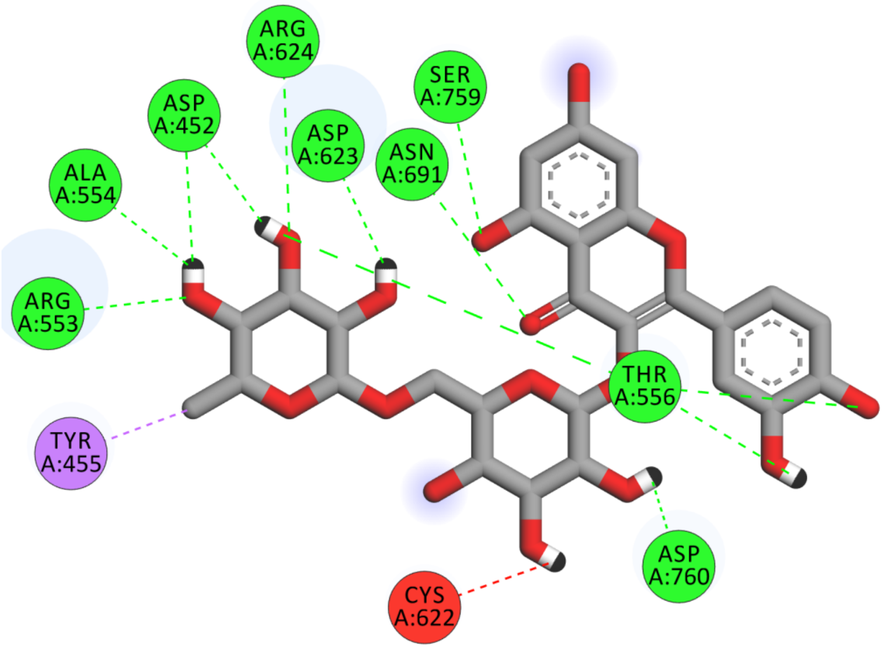

Fig. 21: main interactions observed for quercetin-3-*O*-rutinose (Rutin)-RdRp complex by docking analysis.

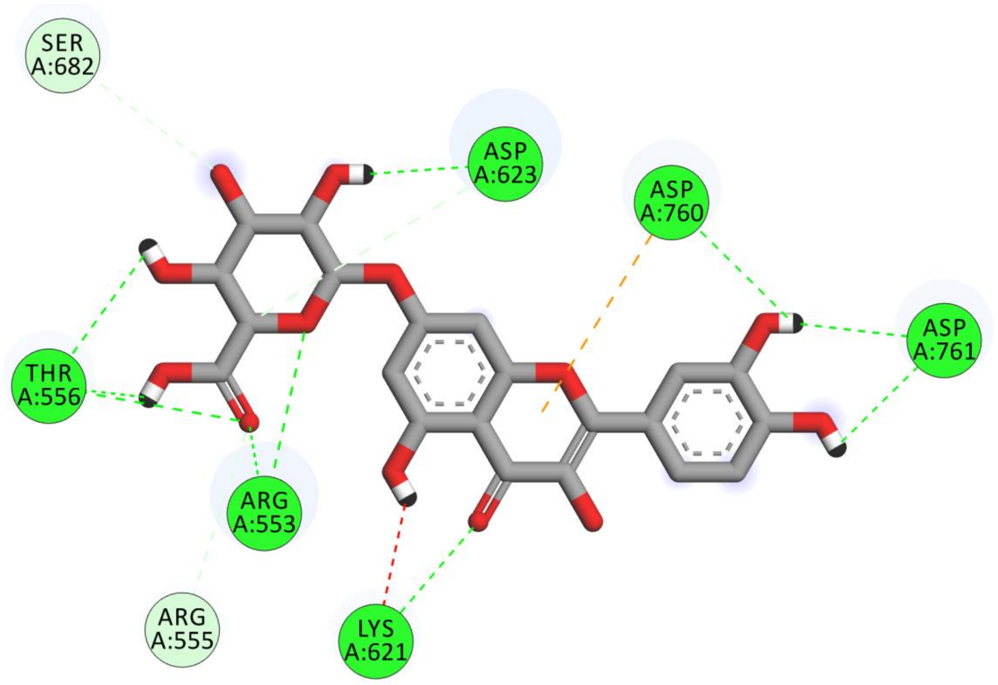

Fig. 22: main interactions observed for quercetin-7-*O*-glucuronide-RdRp complex by docking analysis.

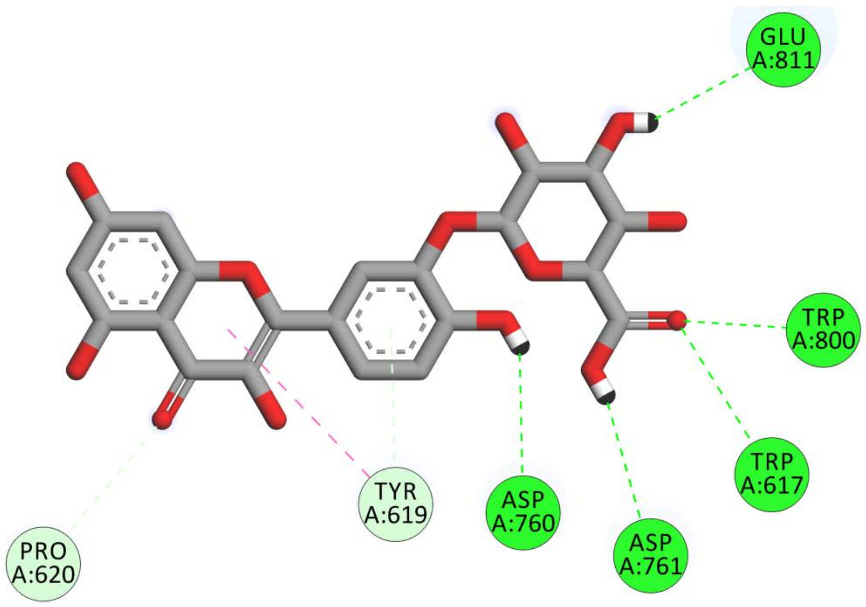

Fig. 23: main interactions observed for quercetin-3'-*O*-glucuronide-RdRp complex by docking analysis.

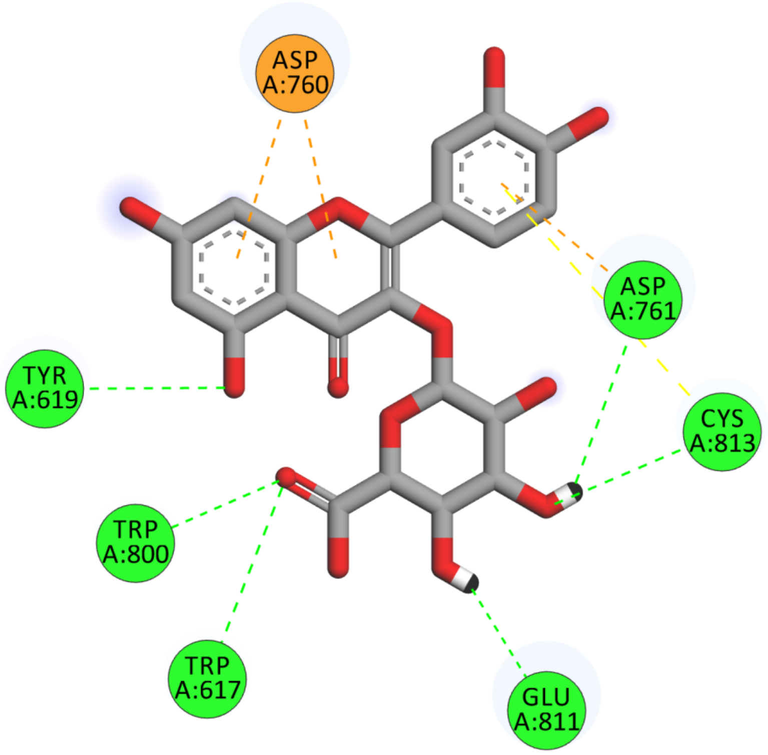

Fig. 24: main interactions observed for quercetin-3-*O*-glucuronide-RdRp complex by docking analysis.

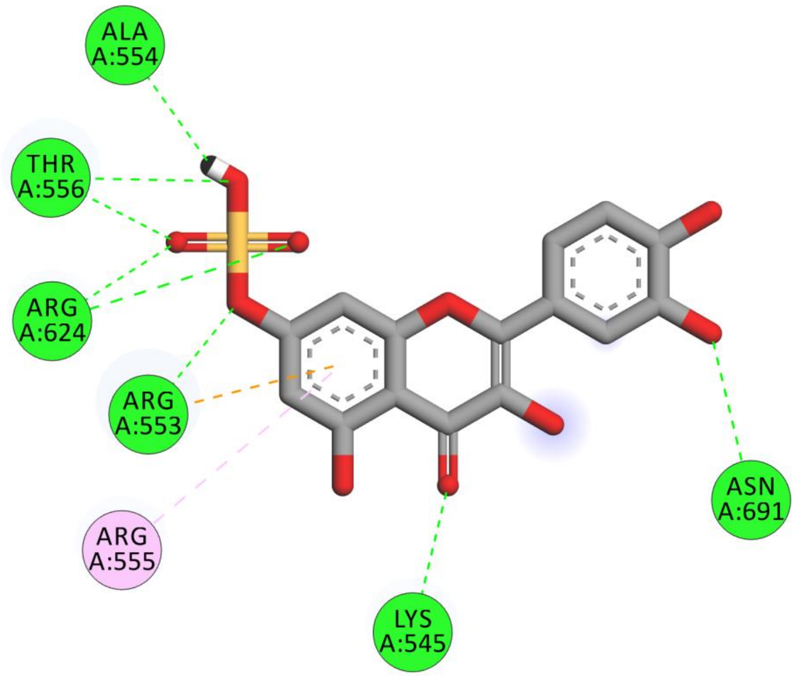

Fig. 25: main interactions observed for quercetin-7-*O*-sulphate-RdRp complex by docking analysis.

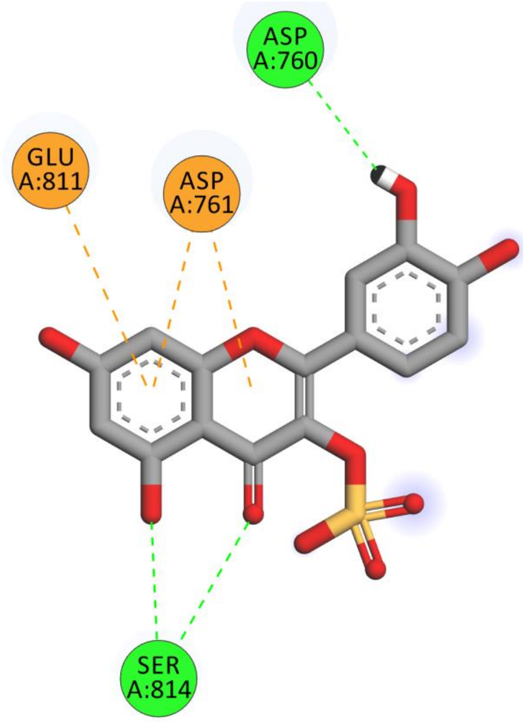

Fig. 26: main interactions observed for quercetin-3-*O*-sulphate-RdRp complex by docking analysis.

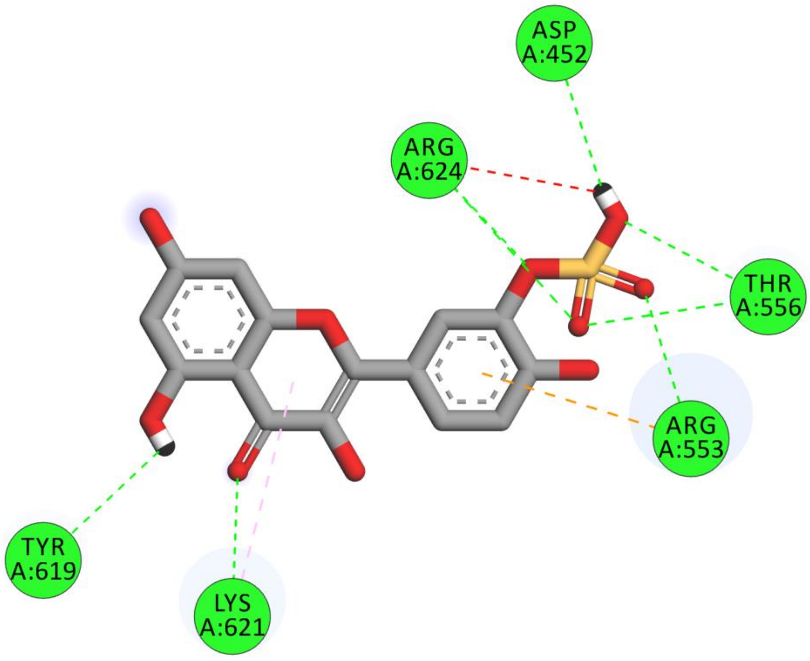

Fig. 27: main interactions observed for quercetin-3'-*O*-sulphate-RdRp complex by docking analysis.

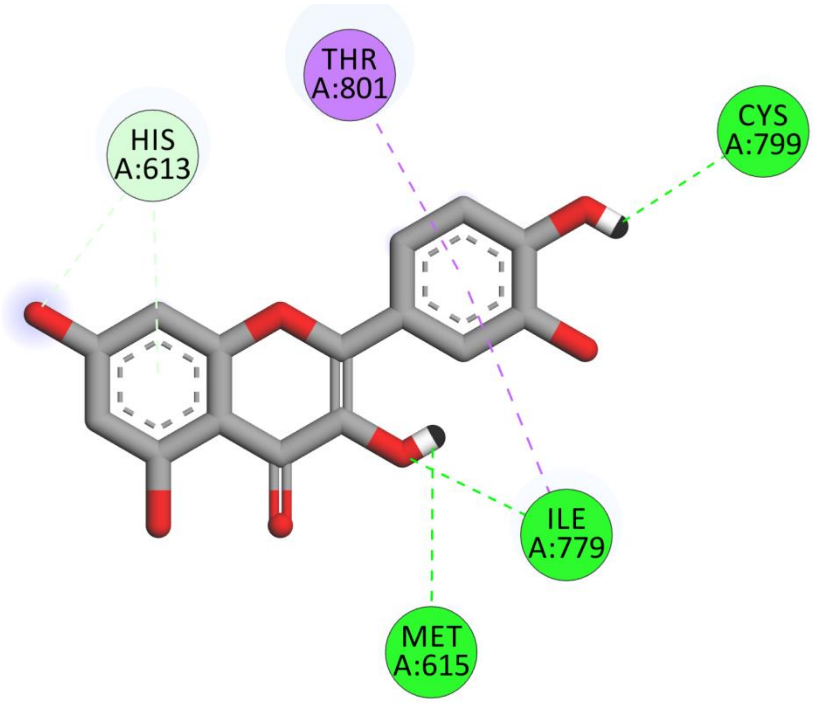

Fig. 28: main interactions observed for quercetin-RdRp complex by docking analysis.

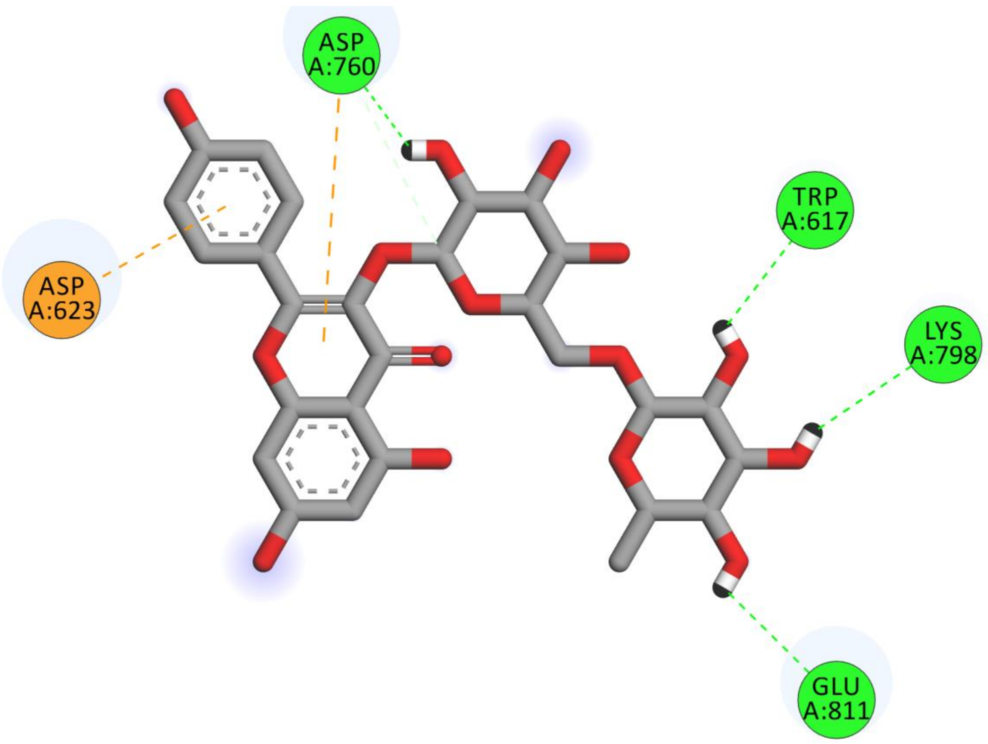

Fig. 29: main interactions observed for kaempferol-3-*O*-rutinose (Nicotiflorin)-RdRp complex by docking analysis.

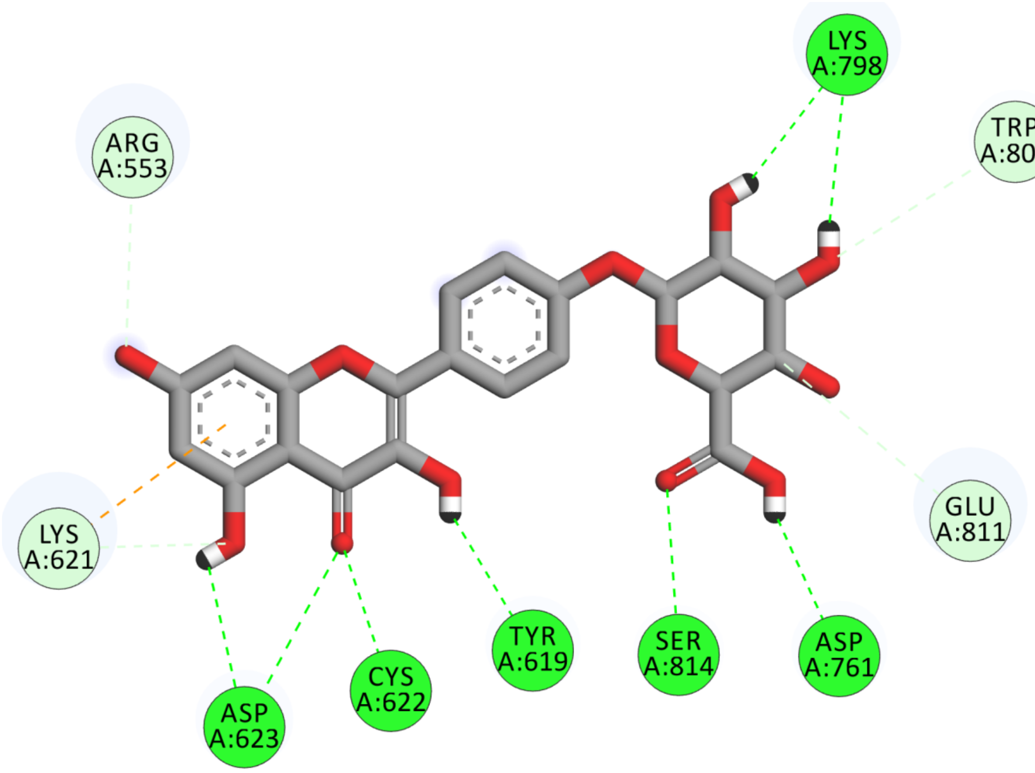

Fig. 30: main interactions observed for kaempferol-4'-*O*-glucuronide-RdRp complex by docking analysis.

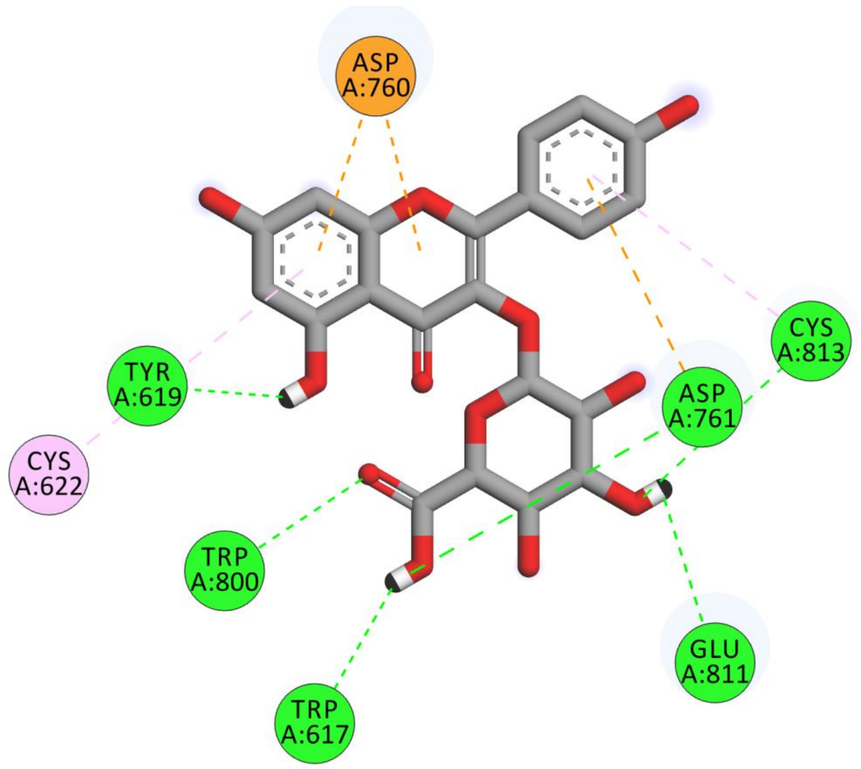

Fig. 31: main interactions observed for kaempferol-3-*O*-glucuronide-RdRp complex by docking analysis.

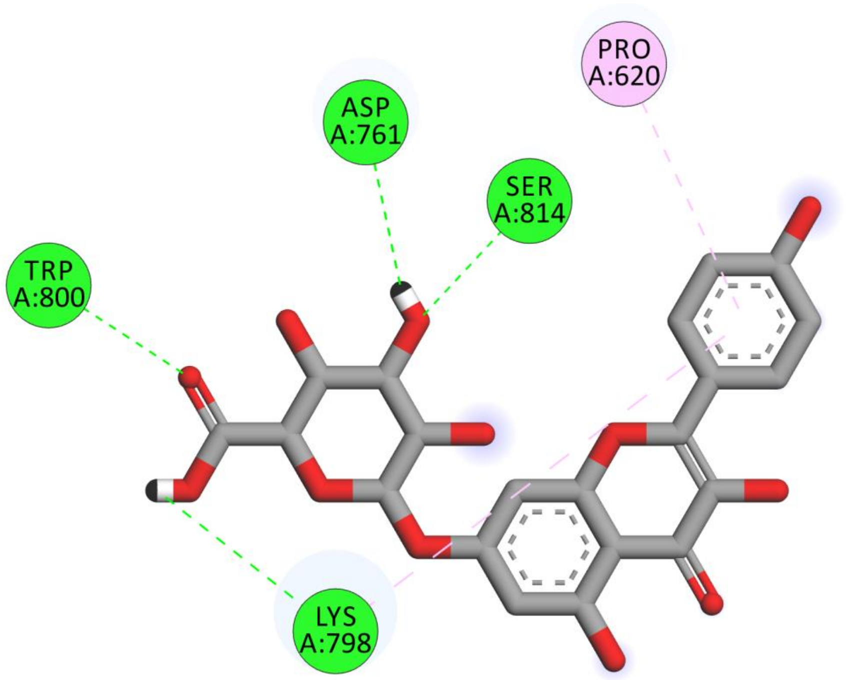

Fig. 32: main interactions observed for kaempferol-7-*O*-glucuronide-RdRp complex by docking analysis.

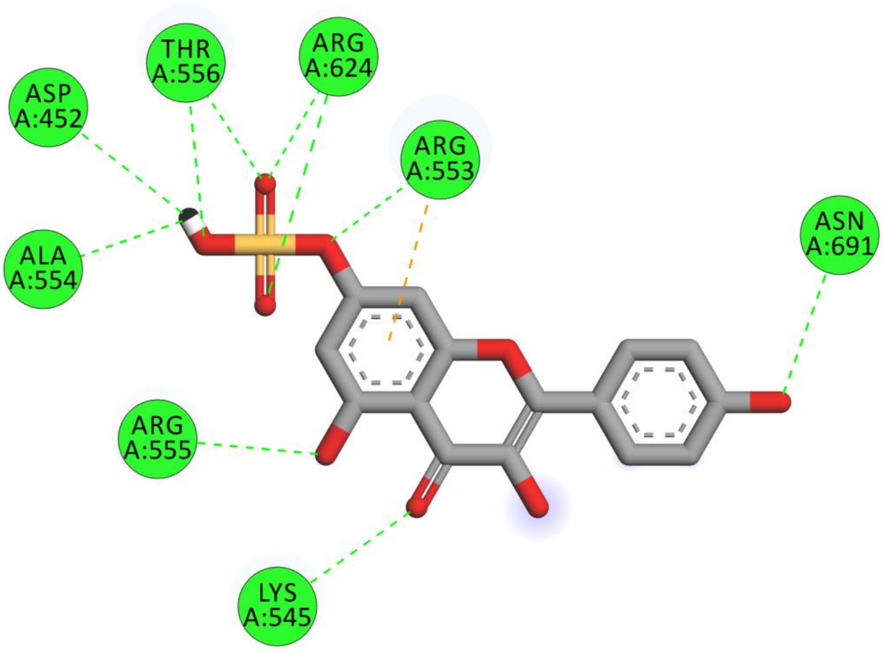

Fig. 33: main interactions observed for kaempferol-7-*O*-sulphate-RdRp complex by docking analysis.

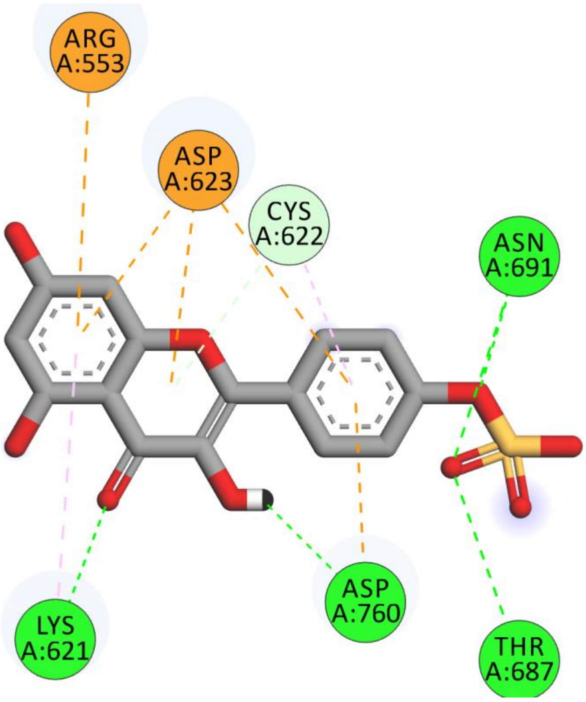

Fig. 34: main interactions observed for kaempferol-4'-*O*-sulphate-RdRp complex by docking analysis.

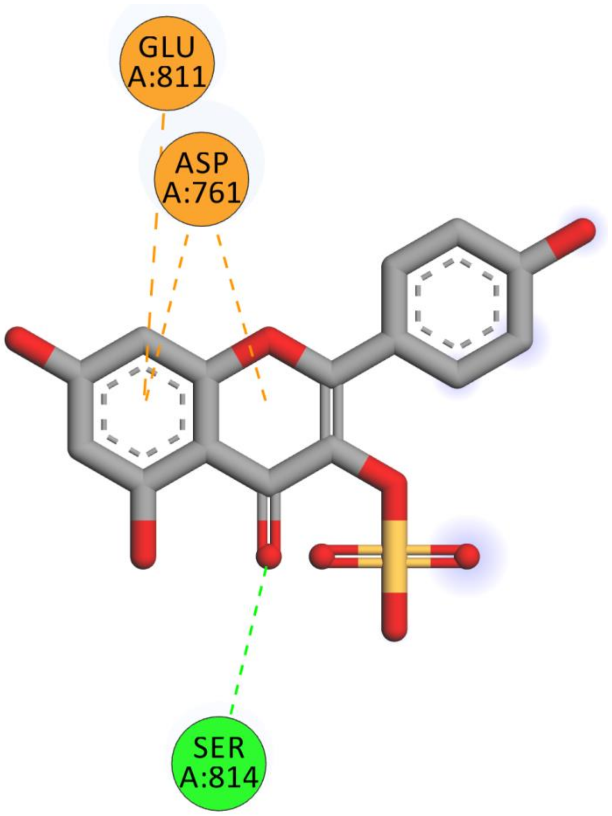

Fig. 35: main interactions observed for kaempferol-3-*O*-sulphate-RdRp complex by docking analysis.

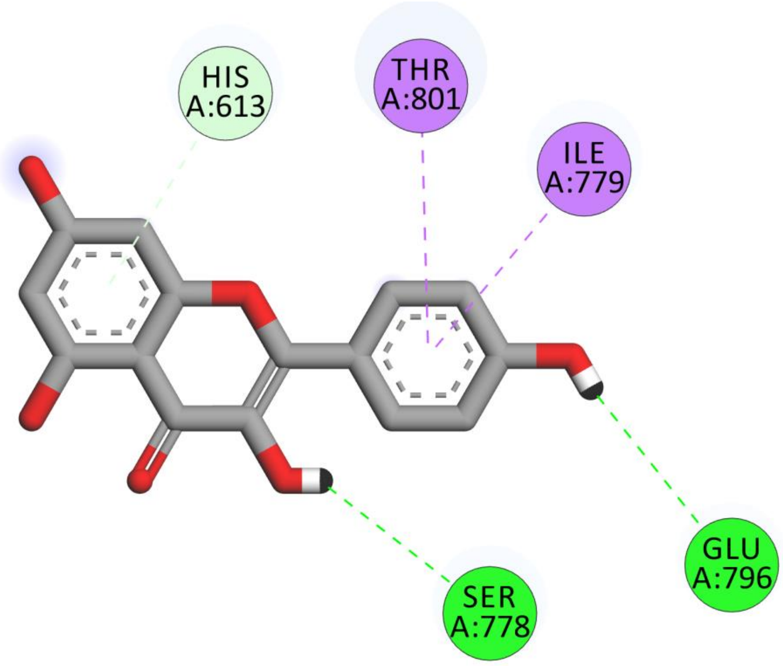

Fig. 36: main interactions observed for kaempferol-RdRp complex by docking analysis.
